# The evolution of gene regulation, the RNA universe, and the vexed questions of artefact and noise

**DOI:** 10.1186/1741-7007-8-97

**Published:** 2010-07-16

**Authors:** Miranda Robertson

## Editorial

In the ten years since the first sequencing of the human genome, much has been made of the need to look to gene regulation, and not gene number or DNA sequence, for the evolution of organismal diversity and complexity - an issue that rose to prominence, with the realization first, that the number of human genes is about the same as the number required to specify a nematode worm; and second, that the DNA of *H. sapiens *is roughly 96% identical to that of the chimpanzee.

But the realization that the secret of evolution lies in changes in gene regulation considerably predates the revelations of genomics. Allan Wilson and colleagues, in a paper published in 1974 [1], drew attention to the simple and striking fact that morphologically homogeneous frog species also have relatively homogeneous karyotypes, whereas mammalian species, which are markedly diverse morphologically, also show major differences in chromosome number and organization; changes in proteins, by contrast, are much the same for both groups. They concluded that genome organization, and by implication gene regulation, is more important for metazoan evolution than protein sequence (and cite earlier publications of EB Ford and Susumu Ohno for the same insight). The following year, Mary-Claire King and Wilson published a more detailed examination of the chromosomal distinctions between human and chimpanzee [2], arguing compellingly, without benefit of high-throughput anything, that changes in the organization of the genome, and not changes in protein-coding sequence, must account for the crucial differences between the two primates.

In those pre-genomic days, the protein data were in large part immunological and electrophoretic; the analysis of genome reorganization depended on chromosome banding patterns (Giemsa banding, not FISH); and almost nothing was known of the mechanism of gene regulation in eukaryotes. The ground between then and now is covered in a recent review by Sean Carroll [3], who acknowledges Emile Zuckerkandl and Eric Davidson as early proponents of the importance of gene regulation in morphological evolution and charts the remarkable history of the development of ideas consequent on the discovery of the homeobox genes, with a strong emphasis on the evolution of *cis*-regulatory elements - that is to say, DNA binding sites for gene regulatory proteins - as the basis for morphological change. The argument is that DNA regulatory elements and the proteins that bind to them, often combinatorially, constitute regulatory networks that can evolve rapidly through changes to the regulatory elements, which are often modular, different modules binding different proteins characteristic of distinct differentiated states of a cell. The gene regulatory proteins can also change, of course, but are generally more highly conserved than their binding sites. Tuch *et al*. [4] have published a short and pellucid overview of the essential points and principles of this schema, in the context of recent evidence on how such regulatory circuits can become rewired in yeast.

In our video Q&A published today [5], John Mattick gives a personal account of his arguments for the view that the regulatory potential of proteins and their binding sites is not sufficient to account for the evolution of complex higher organisms, and explains his case for invoking a largely uncharted universe of regulatory RNAs.

## Beyond regulatory proteins

He puts his points much more eloquently and persuasively than I could, and I will not rehearse them here: so for an elaboration of the argument, and for how the structural properties of RNA lend themselves to exploitation in the regulation of gene expression, or how its functional versatility may contribute to the evolution of cognition, I refer the reader to the interview (which is available as text as well as video).

But a significant part of the basis for his ideas lies in reports over the past several years that most of the genome is transcribed (see especially [6]). Since less than 2% of the human genome, in particular, encodes proteins, this would appear to mean an RNA world on a scale well beyond that of the known world of proteins, and the possibility of a hitherto undreamed of regulatory resource.

The alternative view is that most of the non-coding RNA can be accounted for as technical artefact or transcriptional noise (see [7]).

## Vexed questions

Technical artefact is an issue because much of the evidence for wholesale transcription of the genome derives from tiling array technology, in which labelled cDNAs representing the transcriptome are hybridized to arrays representing the entire genome, and which is susceptible to false positives due to hybridization with imperfectly matched probes. With the more recent development of techniques for high-throughput sequencing of cDNAs (RNA-seq), it has become possible tackle the transcriptome by direct sequencing, eliminating the problem of cross-hybridization and leading to much lower estimates of the proportion of the genome that is transcribed.

The functional significance of the transcripts has been called into question on several grounds: for example, many are rare, or rapidly degraded; and they are generally ill conserved. But these arguments can be reasonably easily turned on their head, and precisely the same properties construed as consistent with, if not indicative of, a regulatory role. The detailed arguments and counter-arguments can be found in reviews by Mattick and colleagues, and by Timothy Hughes and Harm van Bakel from the opposing viewpoint, published last year in *Briefings in Functional Genomics and Proteomics *[7,8].

More recently, Hughes and colleagues have published a paper [9] directly addressing the question of artefact by comparing the results of tiling array experiments and RNA-seq on a range of human and mouse tissues and cell lines, and pursuing the issue of function through an analysis of those transcripts that emerge as valid in the RNA-seq results. They conclude, first, that the great majority of the non-coding transcripts identified in tiling arrays are cross-hybridization artefacts, leaving 12% that are also identified by RNA-seq; and second, that of those, the great majority can be accounted for as unannotated exons of known genes, or introns of known genes, or transcriptional noise due to overrunning polymerases, leaving 2% as non-coding RNA of unknown function. This second point, on the nature and the functional significance or otherwise of the transcripts, is a matter of interpretation, and can no doubt be debated. Nor is the first point exempt from challenge: RNA-seq analysis, like any other genomic analysis, may give different results depending upon how it is done, and rare transcripts, for example, may be missed: a news report [10] on the van Bakel *et al*. paper [9] quotes Philipp Kapranov, whose RNA-seq analysis apparently delivers much higher estimates of non-coding transcription. But there have been other indications of false positives from tiling arrays, and it is difficult to escape the conclusion that the non-coding RNA universe may turn out to be substantially smaller than earlier analyses suggest.

## The evolution of complexity

Where does this leave the issue of how to account for the complexity of higher organisms? (Let us put aside the question of how exactly complexity is defined, on the grounds that we can probably all agree that on any relevant criteria a human being is more complex than a nematode worm.) One implication of the van Bakel *et al*. paper is that there are more exons in the genome than we know about, which would imply more complexity than has yet been tallied in the protein universe. Nor has it been demonstrated by any rigorous computation that combinatorial control of gene expression by protein complexes is insufficient to support the regulatory complexity required to make a human (to which alternative splicing of coding RNAs is likely to make a significant contribution - see for example[11]). However it is clear that even if alternatively spliced and combinatorially interacting proteins were in principle adequate to the task, in practice that is not the sole regulatory resource, and there do indeed exist regulatory RNAs, some quite well understood, others much less well (see [5]). Regulatory RNAs of course also exist in bacteria, where they have been known for 30 years and have a considerable diversity of functions that are much better understood than the more recently discovered eukaryotic ones, and indeed richly illustrate the regulatory modes to which RNA lends itself [12] - a fact that Mattick does not mention in his Q&A for *BMC Biology *but has acknowledged clearly in other publications (see for example [8]). However there is already known to be quantitatively more regulatory RNA in mammals, even without the unexplained non-coding transcripts that have emerged from transcriptomics.

## In biology, the answer is (almost) always yes

The magnitude of the contribution of technical artefact, unannotated coding sequence and transcriptional noise to the reported non-coding transcriptome may not yet be settled, but it would be astonishing if they didn't all contribute. As for whether the evolution of complexity depends on regulatory proteins or regulatory RNAs, the answer is certain to be yes to both. There is much still to be learned about gene regulatory circuits operated by proteins, which will no doubt turn out to include RNA components; and even more to be learned about regulatory RNA. It is the allure and promise of this unexplored territory that Mattick clearly finds irresistible.
